# Data on Indicators used in Southeast Asian nations’ 4th and 5th National Reports to the Convention on Biological Diversity

**DOI:** 10.1016/j.dib.2020.105705

**Published:** 2020-05-15

**Authors:** Xuemei Han, Bruce E. Young, Michael J. Gill, Healy Hamilton, Sheila G. Vergara

**Affiliations:** a NatureServe, 2550 South Clark Street, Suite 930, Arlington, VA 22202, USA; b ASEAN Centre for Biodiversity, Domingo M. Lantican Avenue, Los Baños, Laguna 4031, Philippines; c Fairfax County Department of Management and Budget, 12000 Government Center Parkway, Suite 561, Fairfax, VA 22035

**Keywords:** Biodiversity Indicators, ASEAN, Convention on Biological Diversity, National Reports, Indicator Use

## Abstract

The data presented in this article are related to the research article entitled “Progress on National Biodiversity Indicator Reporting and Prospects for Filling Indicator Gaps in Southeast Asia ” (Han et al., 2020). We examined quantifiable information about biodiversity indicators from the most recent two national reports (i.e., 4th in 2010 and 5th in 2015) to the United Nation's Convention on Biological Diversity (CBD) by the 10-member countries of the Association of Southeast Asian Nations (ASEAN): Brunei Darussalam, Cambodia, Indonesia, Laos, Malaysia, Myanmar, Philippines, Singapore, Thailand and Vietnam. This article presents the number of indicators, their level of development, and detailed lists of indicators for each country, and demonstrates general improvement in indicator use by the highest level of government reporting about implementation of the CBD at the national scale.

Specifications Table

**Table:** 

Subject	Environmental Science (General)
Specific subject area	1. Management, Monitoring, Policy and Law 2. Nature and Landscape Conservation
Type of data	Table
How data were acquired	Literature review and document analysis
Data format	Raw Analyzed
Parameters for data collection	Parties to the United Nations’ Convention on Biological Diversity (CBD) are required to report every 5 years on both the actions implementing the Convention and the effectiveness of these actions (CBD Article 26). These reports represent the highest level of national government reporting on biodiversity. We chose 10 ASEAN Member States to examine their national reports for this study.
Description of data collection	We reviewed the 4th and 5th national reports presented in 2010 and 2015, respectively, to the CBD from the governments of 10 ASEAN Member States (one Southeast Asian country, Timor Leste, is not an ASEAN Member State and is excluded from our analysis), and extracted quantifiable information regarding the use of biodiversity. We identified indicators, counted their numbers, evaluated their level of development, and presented the detailed list of these indicators for each country by Tables included in this paper.
Data source location	Ten member countries of the Association of Southeast Asian Nations (ASEAN): Brunei Darussalam, Cambodia, Indonesia, Laos, Malaysia, Myanmar, Philippines, Singapore, Thailand and Vietnam
Data accessibility	With the article
Related research article	Xuemei Han, Michael J.Gill, Healy Hamilton, Sheila G.Vergara, Bruce E.Young. 2020. Progress on national biodiversity indicator reporting and prospects for filling indicator gaps in Southeast Asia. *Environmental and Sustainability Indicators*, https://doi.org/10.1016/j.indic.2019.100017[1]

## Value of the data

• The Convention on Biological Diversity's (CBD's) adoption of the 2020 Aichi Targets called for the use of indicators to monitor biodiversity and report conservation progress. Unfortunately, appropriate indicators are hard to find in many countries due to a lack of underlying data and limited capacity to generate indicators. National Reports to the CBD represent the highest level of government reporting about implementation of the CBD and contain the most comprehensive biodiversity information available at national scales. As such, they are potentially valuable sources for biodiversity indicators. However, these reports are written by Parties independently, in varying formats, and with extensive contextual information associated in which indicators are presented in different ways. Extracting biodiversity indicators from the National Reports requires delicate data-mining, proper information categorizing, and understanding linkages between indicators and conservation targets; performing this exercise would be a time-consuming activity for general conservation practitioners. The data presented in this article provide complete and explicit lists of indicators used in the 10 Southeast Asian's ASEAN members’ two most recent national reports to the CBD.
• These data can serve as a reference and benchmark for these 10 CBD parties, as well as their neighboring countries while compiling their future national reports.
• These data enable evaluation of biodiversity indicator use, demonstrate the change as well as commonality of indicator uses in Southeast Asia, and thus will assist further research identifying existing indicator gaps and opportunities for indicator use in the region.
• These data can catalyze further measures to improve national-level data coverage and monitoring capacity to generate indicators, facilitating evidence-based policy-making.

## 1 Data Description

Table 1 summarizes the type (i.e., level of development) and number of biodiversity indicators used in 10 ASEAN nations’ 4th (2010) and 5th (2015) National Reports to the Convention on Biological Diversity.

**Table 1:** Type and number of Indicators used in 10 ASEAN nations’ 4th (2010) and 5th (2015) National Reports to the Convention on Biological Diversity.

Country	Indicator type	Number of indicators used in 4th National Report	Number of indicators used in 5th National Report
Brunei	Descriptive	2	2
Brunei	Subnational	0	0
Brunei	Quantitative Baseline	4	9
Brunei	Quantitative Trend	1	1
Cambodia	Descriptive	4	4
Cambodia	Subnational	0	1
Cambodia	Quantitative Baseline	5	2
Cambodia	Quantitative Trend	6	14
Indonesia	Descriptive	0	5
Indonesia	Subnational	5	0
Indonesia	Quantitative Baseline	9	20
Indonesia	Quantitative Trend	9	15
Laos	Descriptive	1	0
Laos	Subnational	1	0
Laos	Quantitative Baseline	10	7
Laos	Quantitative Trend	8	4
Malaysia	Descriptive	1	0
Malaysia	Subnational	1	1
Malaysia	Quantitative Baseline	11	15
Malaysia	Quantitative Trend	6	10
Myanmar	Descriptive	1	4
Myanmar	Subnational	1	1
Myanmar	Quantitative Baseline	13	4
Myanmar	Quantitative Trend	7	10
Philippines	Descriptive	0	4
Philippines	Subnational	6	2
Philippines	Quantitative Baseline	6	8
Philippines	Quantitative Trend	15	22
Singapore	Descriptive	0	0
Singapore	Subnational	0	0
Singapore	Quantitative Baseline	7	7
Singapore	Quantitative Trend	1	3
Thailand	Descriptive	0	0
Thailand	Subnational	3	0
Thailand	Quantitative Baseline	18	8
Thailand	Quantitative Trend	3	18
Vietnam	Descriptive	0	2
Vietnam	Subnational	5	2
Vietnam	Quantitative Baseline	12	14
Vietnam	Quantitative Trend	6	18

Table 2 is the detailed list of biodiversity indicators used in Brunei Darussalam's 4th and 5th National Reports and the type of these indicators.

**Table 2:** List of Indicators used in Brunei Darussalam's 4th and 5th National Reports [4,5].

*Brunei, 4th National Report*	
**Type**	Indicator
Baseline	Area and percent coverage of functional forest classes
Baseline	Bio-ecotypes in different types of forest
Baseline	Total area, species richness of coral reefs on the coastline
Baseline	Species diversity (number of species and endemic species) of plants, mammals, birds, reptiles, amphibians, and fishes
Trend	Law, regulations, and their enactment years
Descriptive	Invasive species
Descriptive	ASEAN Heritage sites
*Brunei, 5th National Report*	

Type	Indicator

Baseline	Area and percent coverage of functional forest classes
Baseline	Distribution of gazetted and proposed forest reserves
Baseline	Bio-ecotypes in different types of forest
Baseline	Marine protected areas
Baseline	Species diversity: number of species recorded during Belait Peat swamp forests survey, including critically endangered and endemic for plants, dragonflies, fishes, amphibians, reptiles, birds, bats, and some mammals (not all categories for all taxa)
Baseline	Total area, species number, and distribution of coral reefs on the coastline
Baseline	Community distribution, number of species, families, genera, and endemism of marine and coastal species, including coral, reef mollusks, seagrasses, shrimp, demersal fishes, marine mammals, and marine turtles
Baseline	Species diversity (number of species and endemic species) of plants, mammals, birds, reptiles, amphibians, and fishes
Baseline	Distribution and identification of critical priority forest habitats for conservation
Trend	Laws, regulations, and their enactment years
Descriptive	Invasive species
Descriptive	Agriculture diversity

Table 3 is the detailed list of biodiversity Indicators used in Cambodia's 4th and 5th National Reports and the type of these indicators.

**Table 3:** List of Indicators used in Cambodia's 4th and 5th National Reports [6,7].

*Cambodia, 4th National Report*	
Type	Indicator
Baseline	Area, number and IUCN category of protected area by major ecosystems
Baseline	Species diversity: number of known species of mammals, birds, reptiles, fishes, amphibians, vascular plants, hard corals, soft corals, and seagrasses
Baseline	IUCN Red List species number and status by taxon (mamma, bird, reptile, amphibians, fish, and plant)
Baseline	Agriculture: number of rice strains
Baseline	Agriculture: percent area of different rice ecosystems
Trend	Forest cover by type (2002, 2006)
Trend	Forest cover (1969, 1993, 1997, 2002, 2005)
Trend	Agriculture: rice production (2003-2007)
Trend	Law and policy enactment years
Trend	Area of aquatic habitats by type (1985-87, 1992-93)
Trend	Mangrove forest area (1993, 1997)
Descriptive	Climate change
Descriptive	Hydrological regime
Descriptive	Invasive species
Descriptive	Genetic erosion issues
*Cambodia, 5th National Report*	

Type	Indicator

Baseline	Area, number and category of protected area by major ecosystems
Baseline	IUCN Red List species number and status by taxon (mammals, birds, reptiles, amphibians, fishes, and plants)
Trend	Protected coverage in 1925, 1957, 1993 and 2014
Trend	Species diversity: number of known species of mammals, birds, reptiles, fishes, amphibians, vascular plants, hard corals, soft corals, and seagrasses in 4th and 5th biodiversity report (2010, 2013)
Trend	Forest cover by type (2002, 2006, 2010)
Trend	Forest cover (1965, 1992, 1996, 2002, 2006, 2010)
Trend	Agriculture: rice production (2008-2012)
Trend	Agriculture: four main crop cultivated area (2003-2012)
Trend	Agriculture: family livestock production (2008-2012)
Trend	Agriculture: family poultry production (2008-2012)
Trend	Aquaculture: Inland and marine capture and aquaculture production (2009-2013)
Trend	Forestry rubber development (2008-2012)
Trend	Forestry: production of logs and processed forest products (2007-2011)
Trend	Laws, regulations, and their enactment years
Trend	International tourist arrivals (1993-2011)
Trend	Tourism monthly trend (2008-2013)
Descriptive	Climate change
Descriptive	Hydrological regime
Descriptive	Invasive species
Descriptive	Genetic erosion issues
Subnational	Water bird species status in Prek Toal Area (2004-2012)

Table 4 is the detailed list of biodiversity Indicators used in Indonesia's 4th and 5th National Reports and the type of these indicators.

**Table 4:** List of Indicators used Indonesia's 4th and 5th National Reports [8,9].

*Indonesia, 4th National Report*	
Type	Indicator
Baseline	Species diversity: number of total and endemic plant, mammal, bird, reptile, amphibian and fish species
Baseline	Genetic diversity: number of accessions, species, and collector institutions in food, agriculture, and livestock sectors
Baseline	Export of forest products (first quarter 2008)
Baseline	Species and number of breeds of wild plants and animals in captivity (2008)
Baseline	Area and coverage of forests (2007)
Baseline	Coverage of coral reefs, mangroves and seagrasses (2008)
Baseline	Number of invasive plant species
Baseline	Number of lakes, rivers, and watersheds
Baseline	River regimes coefficient value (KRS) of several rivers in Indonesia (2005)
Trend	Area of wetland by type (2000, 2007)
Trend	Deforestation rate (1982-2005)
Trend	Emission of CO2 due to forest fires (1997-2007)
Trend	Number and status changes of threatened bird species in 2004, 2007, and 2008
Trend	Coverage of conservation areas and number of conservation management units established (1981-2007)
Trend	Number of breeding activities for protected and unprotected flora and fauna ex-situ conservation (2006-2008)
Trend	Number and size of marine conservation areas (2006, 2007)
Trend	Number and condition of coral reefs (2004-2007)
Trend	Area of mangrove rehabilitation projects (2002-2004)
Subnational	Number and area of swamps in several islands (2007)
Subnational	Condition and area of mangroves in several provinces (2006/7)
Subnational	Burnt area of reported forest fires in several provinces (2004-2008)
Subnational	Number of detected early fire (hot spot) in several areas in Indonesia (2004-2008)
Subnational	Existing and planned botanical gardens in several provinces through 2010

*Indonesia, 5th National Report*	
Type	Indicator
Baseline	Species diversity: number and endemic number of plant, mammal, bird, reptile, amphibian and fish species
Baseline	Genetic diversity: number of accessions, species and collector institutions in food, agriculture, and livestock sectors
Baseline	Number of families and species of marine flora: seagrasses, algae, mangroves, and mangrove associates
Baseline	Number of families and species of marine fauna: Echinodermata, Polychaeta, Crustacea, corals, and fishes
Baseline	Condition and number of species in karst ecosystems
Baseline	Condition and number of species in mangrove ecosystems
Baseline	Location and area of mangrove forests for wildlife protection
Baseline	Location, number, and area of lakes
Baseline	Distribution and area of peatlands
Baseline	Number of plant species in peat forests
Baseline	Condition of major lake ecosystems
Baseline	Area and coverage of forests
Baseline	Population of valuable microbes in studied forest ecosystem
Baseline	Number of invasive species in agriculture, forestry, and fisheries sectors
Baseline	Units and total area of terrestrial and marine nature reserves, wildlife sanctuaries, national parks, nature recreation parks, grand forest parks game reserves, and conservation area management areas (2013)
Baseline	Number of breeding units of wild plants and animals (2013)
Baseline	Number of marine conservation areas by management effectiveness status (2013)
Baseline	Number, location, theme and area of botanical gardens (2014)
Baseline	Protected area and coverage of mangrove forests in each province (2012)
Baseline	Number of elephants, trained elephants, utilized elephants, and elephant users in the Elephant Training Center (2013)
Trend	Extinction rate for local ciliwung and cisadane fish, and crustaceans (1890-2010)

*Indonesia, 5th National Report*	
Type	Indicator
Trend	Number of locations and condition of coral reefs (2008-2013)
Trend	Implementation area of forest and land rehabilitation (2010-2013)
Trend	Documented distribution area of peatlands in Indonesia by region (1978-2005)
Trend	Area of dry primary forest (2000-2009)
Trend	Laws protecting peatland
Trend	Area of forest types (2000, 2009)
Trend	Total conservation area (2011-2007, 2012)
Trend	Number of botanical garden additions and represented ecosystems
Trend	New forest area (2010-2012)
Trend	Marine conservation area (2003-2013)
Trend	Number of orangutans rehabilitated and released by center location (2011-2013)
Trend	Number and trees produced by community nurseries (2010-2013)
Trend	Reforested area by province (2009-2013)
Trend	Reforested area of mangrove peat swamps by province (2009-2013)
Descriptive	Population change and habitat loss for several species
Descriptive	Pollution of air, water and soil
Descriptive	Over-exploited bird and plant species
Descriptive	Climate change
Descriptive	Biodiversity information system

Table 5 is the detailed list of biodiversity Indicators used in Laos’ 4th and 5th National Reports and the type of these indicators.

**Table 5:** List of Indicators used in Laos’ 4th and 5th National Reports [10,11].

*Laos, 4th National Report*	
Type	Indicator
Baseline	Number of ecoregions, national biodiversity conservation areas, and IBAs
Baseline	Number of rice samples in IRRI gene bank
Baseline	Land use area as defined by vegetation types in different slope classes (1992)
Baseline	Estimated number of flowering plant species
Baseline	Distribution and status of NTFP plants
Baseline	Estimated number of mammal, bird, reptile, amphibian, invertebrate, and fish species
Baseline	Forest area by class (2002)
Baseline	Number and area of forest protection conservation areas (2004)
Baseline	Estimated carbon sequestration and dollar value by forest type (2004)
Baseline	Dollar value of industrial products derived from forest resources (2004)
Trend	Protection of aquatic life in stations in the Mekong River Basin (2000-2006)
Trend	Area and percent coverage of NPAs (1993-2008)
Trend	Number of IUCN threatened species as a percentage of global threatened species by taxon (fish, amphibians, birds, mammals, and retiles) (1996-2004)
Trend	Forest coverage and potential (1943, 1960, 1982, 1992, 2002, 2010, 2020)
Trend	GDP and sector shares (2004-3007)
Trend	Area of tree plantations (1976-2008)
Trend	Agriculture production area and output volume of various agriculture commodities (2005-2008)
Trend	Thirteen species that have become recently extinct
Descriptive	Types of non-timber forest products
Subnational	Percent of agriculture farm area in two villages

*Laos, 5th National Report*	
Type	Indicator
Baseline	Number of ecoregions, wetlands, KBAs and their protection
Baseline	Number of identified fish, amphibian, crab and shrimp species, and estimated proportion of the identified to total

*Laos, 5th National Report*	
Type	Indicator
Baseline	Number of IUCN threatened, near-threatened, and all species by taxon (plants, amphibians, birds, mammals, and reptiles)
Baseline	Estimated dollar value of biodiversity in sectors
Baseline	Fish consumption per capita per year (2013)
Baseline	RAMSAR wetland site establishment and the species protected
Baseline	Number of rice samples in IRRI gene bank
Trend	Area and percent coverage of NPAs (1993-2012)
Trend	Forest cover by region and land cover type (1940, 1982, 1995, 2002, 2010, 2015)
Trend	Rubber plantation area (1990, 2007)
Trend	Number of tourists (1990, 2000)

Table 6 is the detailed list of biodiversity Indicators used in Malaysia's 4th and 5th National Reports and the type of these indicators.

**Table 6:** List of Indicators used in Malaysia's 4th and 5th National Reports [12,13].

*Malaysia, 4th National Report*	
Type	Indicator
Baseline	List and number of World Heritage sites, ASEAN Heritage sites, and RAMSAR sites
Baseline	Species diversity: total, endemic and newly discovered species of mammals, birds, reptiles, amphibians, freshwater fishes, marine fishes, invertebrates, plants, and marine organisms
Baseline	Number of genera and species protected under the Wildlife Conservation Act for Mammals, Birds, Reptiles, Amphibians, Insects and Plants (1972 for peninsular Malaysia, 1997 for Sabah, and 1998 for Sarawak)
Baseline	Area of designated protected PRF/PFE forest (2007)
Baseline	Iconic Species: population and locations of tigers
Baseline	Iconic Species: population and protected/other areas for Asian elephant
Baseline	Iconic Species: population and protected /other areas for orange utan
Baseline	Iconic Species: population and locations of proboscis monkey
Baseline	Iconic Species: population and locations of seladang, tapir, and red-banded langgur
Baseline	Number of germplasm collections conserved in seed gene banks
Baseline	Number and list of breeds and major crossbreeds in farm animals
Trend	Area of MPAs (2007, 2008)
Trend	Policies and enactment years (1978, 1998, 2002, 2004, 2005, et al.)
Trend	Forest area by type and category (1990, 2000, 2005, 2007)
Trend	Ecological Footprint (1999-2005)
Trend	Quality of river basins (2000-2007)
Trend	Area of mangrove replanting projects (2005-2008)
Descriptive	Examples and names of invasive species
Subnational	Examples of numbers of species and accessions of ex-situ conservation of indigenous fruit species

*Malaysia, 5th National Report*	
Type	Indicator
Baseline	Area and number of protected areas and marine protected areas
Baseline	Species diversity: number of wild mammals, birds, reptiles, amphibians, freshwater fishes, invertebrates, fungi, mosses, and hard corals
Baseline	Number of vascular plants and endemism by region
Baseline	Number of species assessed by the IUCN and their Red List categories (2009)
Baseline	Forest area and coverage (2012)
Baseline	Area of designated protected PRF/PFE forests (2012)
Baseline	Dollar value of timber exports, the forestry sector, and its contribution to GDP; forestry sector employment (2012)
Baseline	Mountain ranges and their vegetation types

*Malaysia, 5th National Report*	
Type	Indicator
Baseline	Wetland area, number of RAMSAR sites, and significance of selected sites
Baseline	Area, species number, economic value, protection tools for coral reefs
Baseline	Distribution, area, and number of species of mangroves; mangrove areas in protection plans
Baseline	Agricultural genetic resources (number of rice accessions and preserved specimens of insects)
Baseline	Iconic species: population and protection action plans of Asian elephants by region
Baseline	Iconic species: population and protection action plan of orangutans by park
Baseline	Iconic species: population of proboscis monkeys
Trend	Year gazetted for selected parks (1964,1979, 1984)
Trend	Number of established marine protected areas (1970s-2010s)
Trend	Number of genera and species listed and thereby protected under the Wildlife Conservation Act for mammals, birds, reptiles, amphibians, arachnids, insects and gastropods (1972, 2010)
Trend	Laws, policies and their enactment years (1978, 1998, 2002, 2004, 2005, et al.)
Trend	Forest area and protected forest area by region and type, and their distribution (2009-2012)
Trend	Live coral cover percent (2007-2012)
Trend	Agriculture GDP and contribution to total GDP (2011, 2012)
Trend	Iconic species: population, habitat size, and action plans for recovery of Malayan tigers (1950s, 2004, 2020)
Trend	Iconic species: populations of turtle species (1990-2012)
Trend	Iconic species: establishment of turtle sanctuaries and hatcheries (1991-2010)
Subnational	Area and class of permanent reserved forests in Sabah (2009, 2010, 2012)

Table 7 is the detailed list of Indicators used in Myanmar's 4th and 5th National Reports and the type of these indicators.

**Table 7:** List of Indicators used in Myanmar's 4th and 5th National Reports [14,15].

*Myanmar, 4th National Report*	
Type	Indicator
Baseline	Number, area and percent of total land in declared and proposed protected areas
Baseline	Species diversity: number of recorded and endemic species of plants, mammals, birds, and reptiles
Baseline	Number of IUCN threatened and total species of mammals, birds, reptiles, invertebrates, and plants
Baseline	Forest cover by type (2008)
Baseline	Number of species, genera and families protected under law for mammals, birds, and reptiles
Baseline	Permanent forest estates (area and coverage) (2008)
Baseline	Number of rice germplasm evaluated for biochemical traits
Baseline	Number of crop accessions evaluated for biotic and abiotic stresses
Baseline	Germplasm accession and status in gene banks
Baseline	Number of wetland sites in different areas
Baseline	Number and name of domestic animal breeds
Baseline	Number and name of globally threatened wetland bird species
Baseline	Number and name of exported aquarium fish species
Trend	Forest cover (1990-2005)
Trend	Number of established protected areas (1920-2010)
Trend	Laws related to protected areas (1879-2002)
Trend	Livestock production by type (1990-2006)
Trend	List of imported exotic breeds of cattle, pigs, and poultry (1958-1992)
Trend	Marine turtle nesting establishments (1986-2006)
Trend	Releasing of olive ridley sea turtle hatchlings (2000-2008)
Descriptive	Some major endangered wildlife species recorded in NFC
Subnational	Land use change in Bago Yoma (1995-2007)
*Myanmar, 5th National Report*	

Type	Indicator

Baseline	Number of ecoregions and percent coverage of vegetation types
Baseline	Number, area and percent of total land in declared and proposed protected areas
Baseline	Number of IUCN threatened and total species of mammals, birds, reptiles, amphibians, fishes, aquatic invertebrates, and plants
Baseline	Forest cover by type in 2010
Trend	Species diversity: number of recorded and endemic species of plants, mammals, birds, reptiles, amphibians, and fishes (2009 and 2014)
Trend	Number of newly discovered species of plants, mammals, birds, reptiles, amphibians, and fishes (2009 and 2014)
Trend	Management activity in protected areas (2009-2013)
Trend	Forest cover and average annual deforestation (1990-2010)
Trend	Mangrove area by region (1980, 2010)
Trend	Number of recorded reptile and amphibian species (1997, 2000, 2006, 2010)
Trend	Number of migrating spoon-billed sandpipers (2008-2012)
Trend	Crop genetic resources: list of wild crops relatives that have had their habitat destroyed since 1990
Trend	Marine fauna and flora: Catch Per Unit Effort (1980, 2010)
Trend	Number of established protected areas (1920-2014)
Descriptive	Land use change
Descriptive	Illegal wildlife hunting and trade
Descriptive	Invasive species
Descriptive	Climate change vulnerability
Subnational	Number of bird species recorded at selected wetland protected areas (2012, 2013)

Table 8 is the detailed list of biodiversity Indicators used in Philippines’ 4th and 5th National Reports and the type of these indicators.

**Table 8:** List of Indicators used in Philippines’ 4th and 5th National Reports [16,17].

*Philippines, 4th National Report*	
Type	Indicator
Baseline	Number and names of new species discovered supporting KBAs and AZEs in past 5 years (2009)
Baseline	Number of threatened wildlife species by taxa (mammals, birds, reptiles, amphibians, and plants) (2004, 2007)
Baseline	Area of different forest types, and alienable and disposable land (2003)
Baseline	Number of fish, bird, and terrestrial species, and endemic, and threatened fish species
Baseline	Number of species of hard corals, reef fishes, mollusks, seagrasses and algae (2005)
Baseline	Number of marine and freshwater protected areas in different regions, and changes in hard coral and fish abundance, and fish biomass
Trend	Number and area of KBAs and AZEs (terrestrial, freshwater) and the percentage of their area with formal protection (1996, 2006)
Trend	Number and area of terrestrial and marine protected areas (1997, 2008)
Trend	Annual deforestation rate (2000-2005)
Trend	Reforested areas (1976-2006)
Trend	Production of logs, lumber, plywood and veneer (1997-2007)
Trend	Forest fire disturbance area (1996-2006)
Trend	Quantity and value of fish production by type of fishing operation (1996-2007)
Trend	Number of classified water bodies (2006, 2007)
Trend	Pending environment cases in Ph court and law enforcement, green court (2006, 2007)
Trend	Area of mangroves (1919, 1995,2002, 2003, 2008 sources vary)
Trend	Number of existing and proposed marine protected areas (1995, 1997, 2000, 2007)
Trend	Names and number of documented indigenous knowledge systems and practices, locations and tribes (2005-2008)
Trend	Department of Environment and Natural Resources budget allocation (1987-1999)
Trend	Number of forestry programs and forest management holders (2001-2005)
Trend	Number and areas of approved Certificates of Ancestral Domain Titles and Certificates of Ancestral Land Titles (2002-2008)
Subnational	Population sizes of indicator species in certain areas of – whale sharks, humpback whales, and Irrawaddy dolphins
Subnational	Complete nests and egg production of olive ridley sea turtles in Bataan and Zambales (2004-2009)
Subnational	Number of complete nesting and egg production per year in the Baguan Island Marine Turtle Sanctuary (1984-2007)
Subnational	User fee income in Gilustongan Island Marine Sanctuary (1998-2008)
Subnational	HIETA Income from ecotours (2007-2008)
Subnational	(Ecotourism related) PIDWWO net income (2003-2008)

*Philippines, 5th National Report*	
Type	Indicator
Baseline	Number and area of KBAs (terrestrial, freshwater and marine), and overlap with protected areas
Baseline	Numbers of all, endemic, and threatened fish species
Baseline	Numbers of species of hard corals, reef fishes and mangroves
Baseline	Numbers of marine protected areas, and their protection status
Baseline	Number and percent of provinces, cities and municipalities that are located along coastal areas and affected by the changes in coastal and marine ecosystems
Baseline	Number and percent of river basin master plans completed (2013)
Baseline	Integrated Protected Area Fund collection from top-earning protected areas (2012)
Baseline	Number of developed national strategies and action plans addressing environmental concerns
Trend	Number and area of terrestrial and marine protected areas (2008, 2013)

*Philippines, 5th National Report*	
Type	Indicator
Trend	Number of new species discoveries by taxon (mammals, birds, reptiles, amphibians, and plants) (2005-2012)
Trend	Number of threatened wildlife species by taxon (mammals, birds, reptiles, amphibians, and plants) (2006-2013)
Trend	Philippine eagle sightings in the wild from the DENR site monitoring program (2005-2013)
Trend	Area of forest land classification (2005-2012)
Trend	Forest cover by forest types (2003, 2010)
Trend	Reforested areas (2005-2012)
Trend	Forestry revenues from timber harvest and non-timber forest products (2011, 2012)
Trend	Performance of agricultural industries and subsectors (2012,2013)
Trend	Quantity and value of fish production by type of fishing operation (1996-2012)
Trend	Biochemical oxygen demand for 19 priority rivers (2003,2005, 2009, 2012)
Trend	Dissolved oxygen for 19 priority rivers (2003,2005, 2009, 2012)
Trend	Number, names, and supporting threatened species in proclaimed critical habitats of wetlands, and legal enactment times (2007-2012)
Trend	Significant Writs of Kalikasan issued by Philippine courts (2010-2014)
Trend	Area of mangroves (2003-2012)
Trend	Confiscated wildlife and cases filed in court (2009-2013)
Trend	Extreme weather events related to global warming and their damage in dollar value and people affected (2009-2013)
Trend	Number of classified caves for cave management and protection (2012, 2014)
Trend	Number of industries/firms awarded with DENR official seal for environmental performance as protection incentives (2009-2014)
Trend	Proportion of population with access to safe water supply (1990, 2008, 2011, 2015)
Trend	Proportion of households with sanitary toilet facilities (1990, 2008, 2011, 2015)
Trend	Proportion of households with access to secure tenure (1990, 2010)
Descriptive	Lake pollution
Descriptive	Urban biodiversity
Descriptive	Invasive species impact
Descriptive	Awareness of biodiversity
Subnational	Tamaraw population in Mts. Iglit-Baco National Park 2000-2013
Subnational	Species richness change in reef sites

Table 9 is the detailed list of biodiversity Indicators used in Singapore's 4th and 5th National Reports and the type of these indicators.

**Table 9:** List of Indicators used in Singapore's 4th and 5th National Reports [18,19].

*Singapore, 4th National Report*	
Type	Indicator
Baseline	Distribution of 22 nature sites
Baseline	Number of species newly discovered, new records, and rediscoveries from NParks Natural Areas Surveys Project (2007)
Baseline	Species diversity: numbers of species of vascular plants, ferns and fern allies, fungi, bryophytes, mammals, birds, reptiles, amphibians, freshwater fishes, butterflies, dragon flies, and hard corals (2009)
Baseline	Distribution of mangrove forests
Baseline	Distribution and area of fringing and patch reefs
Baseline	Species diversity: number of recorded marine and freshwater species by taxa (2009)
Baseline	Types and number of managed terrestrial habitats
Trend	Distribution of changes in greenery cover from remote sensing images (1986, 2007)
*Singapore, 5th National Report*	

Type	Indicator

Baseline	Number and names of invasive/exotic species

*Singapore, 5th National Report*	
Type	Indicator
Baseline	Number of species newly discovered, new records, and rediscoveries since the Comprehensive Marine Biodiversity Survey in 2010
Baseline	Number and description of possible extirpated species
Baseline	Singapore Index on Cities' Biodiversity (with comprehensive and highly integrated input data)
Baseline	Number and names of native plants propagated since 2010
Baseline	Distribution of improved nature areas
Baseline	Number of marine invertebrate species by taxon
Trend	Area and percent of Singapore's land requirements by land use type (2010, 2030)
Trend	Species diversity: number of species by taxon and change from the 4th national report (2008, 2015)
Trend	Population of the vulnerable banded leaf monkey (1990, 2008)

Table 10 is the detailed list of biodiversity Indicators used in Thailand's 4th and 5th National Reports and the type of these indicators.

**Table 10:** List of Indicators used Thailand's 4th and 5th National Reports [20,21].

*Thailand, 4th National Report*	
Type	Indicator
Baseline	Number of ecoregion and forest types
Baseline	Species diversity: number of plants, vertebrates, invertebrates, fishes, floral species, and seagrasses
Baseline	Number of threatened species and total species of amphibians, birds, fishes, reptiles, mammals, and plants
Baseline	Number of plant species in each IUCN category by taxon
Baseline	Poaching and deforestation (2004)
Baseline	Area of agricultural land for rice, rubber, cassava, fruit tree, oil palm and vegetables (2006)
Baseline	Area and coverage of mountain ecosystems
Baseline	Number of Important Plant Areas by type of area (2009)
Baseline	Number of Important Plant Areas under various threats (2009)
Baseline	Number of crop species collected at national crop research institute
Baseline	Number and names of invasive species by status
Baseline	Area of swamp forest and percent in very poor status
Baseline	Area, number of communities, distribution, and supported number of fishes, shrimps, crabs, and sea slugs in coral reefs
Baseline	Number of accessions of rice, durian, mango, lichee, and longan
Baseline	Number of wild animals: elephants, wild buffalos, tigers, guars and bantengs, kuprey, eld's deer, and Java rhinoceroses (2004)
Baseline	Area and number of plant and animal species in mangrove forest
Baseline	Area of seagrass beds and number of supported fishes and invertebrate species
Baseline	Estimated number of large marine animals: dugongs, Irrawaddy dolphins, bottlenose dolphins, spotted dolphins, striped dolphins, spinner dolphins, and sharks
Trend	Laws, policies and enactment years
Trend	Coverage of forests (1961, 2005)
Trend	Number of biodiversity-related study and research projects (1997-2008)
Subnational	Number of crop wild relative species in several provinces
Subnational	Number of plant and animal species found in agricultural ecosystems in 5 regions
Subnational	Percent of coral reefs by condition category around Koh Samui and other islands in Suratthani province (2007)
*Thailand, 5th National Report*	
Type	Indicator

Baseline	Number of ecoregion and forest types
Baseline	Species diversity: number of plants, vertebrates, invertebrates, fishes, endemic fishes, corals, and seagrasses

*Thailand, 5th National Report*	
Type	Indicator
Baseline	Number of species in each IUCN category by taxon (mammals, birds, reptiles, amphibians, and fishes) (2013) and plants (2014)
Baseline	Number of new, rare and endemic species and new localities that have threatened status
Baseline	Number of plant species listed as medicinal plants used in Thai traditional medicine
Baseline	Illegal cutting of rosewood: number of cases, arrests, and charges, and number of species seized (2014)
Baseline	Area and percentage of wetland types by region
Baseline	Seagrass sources by region
Trend	Number of total and threatened species of amphibians, birds, fishes, reptiles, and mammals (1996, 2005, and 2013)
Trend	Area and coverage of forests (1973-2013)
Trend	Forest encroachment by region (2012-2014)
Trend	Agriculture land (1962, 2011)
Trend	Number of fishing vessels and catch per hour (1961, 2011)
Trend	Net weight and dollar value of rice export (2007-2013)
Trend	Export value of traditional medicines and herbs (2008-2013)
Trend	Export value of agricultural products, agro-industry products and national GDP (2003-2013)
Trend	Aquaculture area and production of marine shrimp, and area of mangrove forests (1993-2014)
Trend	Percent of wetlands lost (2009, 2013)
Trend	Area of mangroves (2009, 2013)
Trend	Area of coral reefs before and after bleaching (2010, 213)
Trend	Number of animals in wildlife trafficking (2011-2013)
Trend	Weight of illegally traded ivory (2008-2012)
Trend	Dalbergia wood smuggling (2006-2013)
Trend	Fish catch capacity by water source (2002-2011)
Trend	Fishing effort (catch weight and number of vessels) by water source (2002-2011)
Trend	Pollution: weight of garbage, waste water (2010, 2012)

Table 11 is the detailed list of biodiversity Indicators used in Vietnam's 4th and 5th National Reports and the type of these indicators.

**Table 11:** List of Indicators used in Vietnam's 4th and 5th National Reports [22,23].

*Vietnam, 4th National Report*	
Type	Indicator
Baseline	Number of plant species in different types of agricultural use (2005)
Baseline	Number of protected areas by type, and other internationally recognized protected areas (2006)
Baseline	Recently discovered species in past two decades (2005)
Baseline	Number of Species in Vietnam and in the world by taxon (terrestrial, freshwater, and marine; plant, microbial, and animal) (2004)
Baseline	Number of livestock breeds
Baseline	Number of proposed and recognized wetland management areas
Baseline	Number and area of proposed and declared marine protected areas
Baseline	Number of species and seeds preserved by gene resource conservation activities
Baseline	Number of species in different categories of the Vietnam Red Book 2007 by taxon
Baseline	Percent of coral coverage by status category in investigated sites (2005)
Baseline	Revenue of national and international wildlife trade that transits Vietnam (2002)
Baseline	Number of invasive species by risk type (2005)
Trend	Area of mangrove forests and their loss rate (1943, 1990, 2005)
Trend	Area and coverage of forest by type (natural vs. planted) (1990-2006)
Trend	Area of agricultural land (1990, 2002)

*Vietnam, 4th National Report*	
Type	Indicator
Trend	Percent area and percent indigenous species loss due to introduction of new species and invasive alien species by species group (1970-1999)
Trend	Estimated needs for some wood products (2005-2020)
Trend	Recent legal documents related to biodiversity (1997-2007)
Subnational	Some medicinal herb gardens and numbers of species preserved
Subnational	Area of mangrove forests and shrimp farms in Ca Mau and Tra Vinh (1965, 2001)
Subnational	Number of sites and percent of decrease in coral cover in some coastal marine areas (1993-2002)
Subnational	Distribution of mangrove and cajuput forest coverage in Ngoc Hien district, Ca Mau province in 1965 and 2001
Subnational	Heavy metal waste in the environment and animal tissues in Ha Long Bay-Bai Tu long

*Vietnam, 5th National Report*	
Type	Indicator
Baseline	Number and area of wetland sites by region (2009)
Baseline	Number of protected areas by type
Baseline	Number and areas of marine protected areas (2010)
Baseline	Area and coverage of forest by type and region (2010)
Baseline	Number of visitors to national parks (2011)
Baseline	Income from tourism in national parks (2011)
Baseline	Estimate of carbon storage in natural forests by region
Baseline	Land conversion: percent of agricultural land lost to urban and industrial sites (2010)
Baseline	Number of invasive exotic species by taxon (2013)
Baseline	Distribution of population by region
Baseline	Pollution: percent of licensed industrial parks having centralized wastewater treatment systems
Baseline	Pollution: fertilizer use and pollutants in rivers
Baseline	Number of natural habitats, protected areas, and KBAs that would be affected by climate change
Baseline	Number, area, and status of types of centers for ex-situ plant conservation
Trend	Number of species in each National Red List assessment category by taxon (1996, 2007)
Trend	Number of deforestation violations (2008-2012)
Trend	Area and coverage of forest by type (natural vs. planted) (1990-2012)
Trend	Timber volume (2006, 2010)
Trend	Dollar value of agricultural production by sector (planting, breeding, and service) (2006-2012)
Trend	Biomass of pelagic fishery landings (2011-2013)
Trend	Live coral coverage by monitoring site (1994-2008)
Trend	Percent decrease of seagrass habitat by site (time varies with site)
Trend	Area of mangroves (1943-2012)
Trend	Land conversion: natural forest to rubber plantation by site (2008, 2014)
Trend	Area of surface water (salty/brackish and fresh) used for aquaculture 2007-2010
Trend	Area of forest land converted for infrastructure development (2007-2012)
Trend	Documented and forecasted demand for some major timber products (2005-2020)
Trend	Volume of timber confiscated by year for normal timber and valuable timber (2007-2012)
Trend	Number of wildlife trafficked (2008-2012)
Trend	Number of flash floods (1990-2010)
Trend	Stock of fish by fish type and decline rate (2000-2005)
Trend	Number and names of institutes participating in agricultural genetic resources and their year of foundation (1989-2000)
Descriptive	Overexploitation of genetic resources
Descriptive	Inland water fragmentation
Subnational	Estimates of mangrove carbon stocks in Kien Giang (2010)
Subnational	Land conversion: area of coastal sandy ecosystem loss in Ha Tinh and Ninh Thuan

## 2 Experimental Design, Materials, and Methods

Parties to the CBD are required to report every 5 years on both the actions taken toward implementation of the Convention and the effectiveness of these actions (CBD Article 26). These national reports represent the highest level of government reporting about implementation of the CBD at the national scale.

We extracted quantifiable information regarding the use of biodiversity indicator by the governments of 10 ASEAN Member States (one Southeast Asian country, Timor Leste, is not an ASEAN Member State and is excluded from our analysis) in their 4th and 5th national reports to the CBD, presented in 2010 and 2015, respectively. We identified indicators, counted their numbers, evaluated their level of development, and presented the detailed list of these indicators for each country.

The 10 member countries of the Association of Southeast Asian Nations are Brunei Darussalam, Cambodia, Indonesia, Laos, Malaysia, Myanmar, Philippines, Singapore, Thailand and Vietnam. We downloaded the most recent two national reports (i.e., 4th submitted in 2010 and 5th submitted in 2015) to the CBD by these countries’ national government [available at 2 and 3 respectively], and extracted the quantifiable information on biodiversity indicators.

We defined indicators as any descriptive or quantitative metric that was meant to convey information about a biodiversity issue. Some countries reported the trend in gross domestic product and human population as pressures, but we do not count these as specific indicators for this dataset because they provide contextual information and do not necessarily lead to biodiversity threats. In each report we counted the number of indicators presented, as well as their level of development, defined as whether they represent nonquantitative descriptive statements, quantitative baseline (i.e., snapshot data resulting from a single measurement), or quantitative trends measured in multiple years. In some cases, quantitative baseline or trend data were presented for a region of the country or specific sites instead of the entire country; we categorized these cases as subnational indicators.

Table 1 summarizes the type (i.e., level of development) and number of indicators used in the 10 ASEAN nations’ 4th (2010) and 5th (2015) National Reports to the Convention on Biological Diversity. Table 2-11 are the detailed list of Indicators used and their level of development in each country's 4th and 5th National Reports.

## Declaration of Competing Interest

The authors declare that they have no known competing financial interests or personal relationships which have, or could be perceived to have, influenced the work reported in this article.

## Appendix Supplementary materials

Image, application 1
